# IoT Botnet Attack Detection Based on Optimized Extreme Gradient Boosting and Feature Selection

**DOI:** 10.3390/s20216336

**Published:** 2020-11-06

**Authors:** Mnahi Alqahtani, Hassan Mathkour, Mohamed Maher Ben Ismail

**Affiliations:** Computer Science Department, College of Computer and Information Sciences, King Saud University, Riyadh 11543, Saudi Arabia; mathkour@ksu.edu.sa (H.M.); mbenismail@ksu.edu.sa (M.M.B.I.)

**Keywords:** IoT botnet attacks, Fisher score method, feature selection, genetic-based extreme gradient boosting model

## Abstract

Nowadays, Internet of Things (IoT) technology has various network applications and has attracted the interest of many research and industrial communities. Particularly, the number of vulnerable or unprotected IoT devices has drastically increased, along with the amount of suspicious activity, such as IoT botnet and large-scale cyber-attacks. In order to address this security issue, researchers have deployed machine and deep learning methods to detect attacks targeting compromised IoT devices. Despite these efforts, developing an efficient and effective attack detection approach for resource-constrained IoT devices remains a challenging task for the security research community. In this paper, we propose an efficient and effective IoT botnet attack detection approach. The proposed approach relies on a Fisher-score-based feature selection method along with a genetic-based extreme gradient boosting (GXGBoost) model in order to determine the most relevant features and to detect IoT botnet attacks. The Fisher score is a representative filter-based feature selection method used to determine significant features and discard irrelevant features through the minimization of intra-class distance and the maximization of inter-class distance. On the other hand, GXGBoost is an optimal and effective model, used to classify the IoT botnet attacks. Several experiments were conducted on a public botnet dataset of IoT devices. The evaluation results obtained using holdout and 10-fold cross-validation techniques showed that the proposed approach had a high detection rate using only three out of the 115 data traffic features and improved the overall performance of the IoT botnet attack detection process.

## 1. Introduction

**Figure 1:**
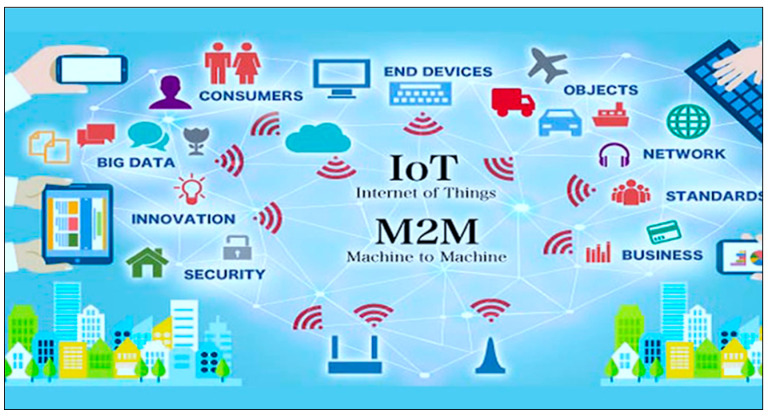
Machine-to-machine (m2m) and machine-to-human (m2h) communication in IoT (https://www.peerbits.com/blog/difference-between-m2m-and-iot.html).

The Internet of Things (IoT) encompasses almost everything one could imagine in today’s world [1], ranging from body sensors to cloud computing and much more. As such, integration is continuously occurring through IoT. It pervasively connects machines, humans, and networks into a complex set of distributed systems [2]. The machine-to-machine communication and machine-to-human communication ability of IoT increases the human life experience to a new level, as depicted in Figure 1. Some examples are smart homes [3], smart grids [4], smart cities [5], and the Industrial Internet of Things (IIoT) [6,7,8].

With regard to research prospects, IoT sensors have been developed to be adaptive and intelligent devices due to the immense potential applications and technologies they are being integrated into. Although there is no formal definition of IoT from an engineering perspective, IoT can be presented as a network of many things, each having a computing system (i.e., CPU, memory, power source) and a communication interface, such as radio or Ethernet [9]. These devices are connected to the Internet and can be identified by their unique address [10]. IoT has a broad set of applications and has proven to be useful for society. However, there are some security concerns related to IoT [11]. At times, IoT devices are left unattended while they are continuously monitoring in an environment or place, which is a considerable security and privacy concern [12]. There are numerous security actions that can be deployed to protect the systems of IoT networks and devices. However, experts have concluded that it is not possible to completely avoid all kinds of attacks [13,14].

In fact, having invulnerable systems is extremely costly. In addition, control measures may be counterproductive and affect the system performance [14]. In other words, it is impractical to individually secure each and every device in the IoT infrastructure because of the huge scale of the networks. Additionally, the data can be constantly observed as it flows throughout the network; hence, network-based security can be implemented. In contrast to device security, network security can be easily adapted to the IoT environment with minor subsequent modifications.

Intruder intervention can be limited by registering devices to the network [15]. Anomaly detection can be implemented in order to identify any change in the events or items within the IoT network. In the case of network traffic, all incoming and outgoing traffic can be closely monitored to give full control over the behavior of the network traffic. The owner of the device is alerted in case an unwanted or unreliable change in behavior is detected.

The network traffic attacks in IoT systems can cause unusual behavior from IoT sensors and devices or even data loss for the end users [16,17,18,19]. In fact, IoT devices and wireless networks are widely targeted by several types of attacks. For example, researchers conducted a specific intrusion attack experiment for a car crash to prove the effects of a real attack [16]. In addition, the attacks on medical network protocols have revealed another vulnerability that may cause problems to patients [17,18]. In the past few decades, a number of research works have been proposed for different types of networks. Some of these were for mobile ad hoc networks [19,20,21]. Others were related to wireless sensor networks (WSNs) [22,23,24], cloud computing [25], cyber–physical systems [26], and wide area networks (WANs) [27,28,29]. The recent spread of IoT devices has introduced new threats such as botnet attacks [30]. Such attacks appear to compromise the victim devices, and the attacks can be coordinated. A botnet can be defined as illegal remote control of a host. The compromised IoT devices are controlled by attackers to perform malicious activities [31].

In the real world, huge damage can be caused by botnets, such as the Mirai attack, which affected almost 1000 closed circuit television (CCTV) cameras in 2016 [32]. In this case, the distributed denial-of-service (DDoS) attack operated by flooding the CCTV with HTTP get requests [33]. Several studies have reported that some IoT devices, such as baby monitors, stoves, and refrigerators, have been infected by intrusion attacks [34,35]. Another study illustrated that botnet attacks were used to compromise the power grid in South Africa by switching stoves to their maximal power for four hours. Due to these constraints and limitations, various studies have reported that detecting threats to IoT devices is a challenging matter that requires special and intelligent intrusion detection system (IDS) tools that must be adapted over IoT application layers [36]. The need to enhance the security of IoT devices has motivated researchers to design host-based IDS methods to prevent such attacks. In particular, the authors in [31,36] proposed the detection IoT botnet attacks using anomaly-based systems. They designed their technique based on the typical behavior of IoT devices. Specifically, any deviation in the IoT device behavior is considered a malicious attack. They reported that this approach is effective in detecting attacks, showing a low positive rate after testing it on two IoT botnets, Mirai and Bashlite [37]. Other researchers tried to place IDS into physical objects by designing optimized lightweight algorithms to match attack signatures and packet payloads [38,39]. A lightweight method was also used to monitor node energy consumption and minimize the resources required for intrusion detection [39]. A new framework for detecting different routing attacks for low-power and low-loss networks, including sinkhole attacks, wormhole attacks, and selective forwarding attacks, was proposed in [40]. 

Cho et al. [41] introduced a detection solution based on botnet attacks by monitoring the data traffic between IoT hosts and networks. The authors proposed the use of an IDS analysis engine in a powerful dedicated host, where selected sensors send data to the engine. Recent studies on IDS IoT host-based methods have proposed an optimized machine learning technique based on selected features of malicious attack behaviors [42]. Diro and Chilamkurti [43] outlined a host-based approach using deep learning as a novel intrusion detection technique for the IoT context, with promising results. Cruz et al. [44] stated the necessity of IoT middleware for implementing IDS intelligence-based and decision-making mechanisms to address IoT resource limitations. Furthermore, the adoption of the deep learning approach using a recurrent neural network (RNN) proved to be efficient in detecting IoT malware [45]. Unfortunately, regarding recent research applying machine learning in IDS of IoT, there is no work that has presented an in-depth view of the application of machine learning in the context of IoT host-based intrusion detection [42].

Due to the need for an efficient and effective solution for detecting botnet attacks in resource-constrained IoT devices, we propose an IoT botnet attack detection approach using a Fisher-score-based feature selection method with a genetic-based extreme gradient boosting (GXGBoost) model. The Fisher score is a representative filter-based feature selection method that plays an effective role in dimensionality reduction by minimizing within-class distance and maximizing between-class distance. The GXGBoost is an optimized model that uses the extreme gradient boosting (XGBoost) method for classification and the genetic algorithm for selecting optimal values of XGBoost’s parameters and increasing the accuracy of the minority classes without affecting the overall accuracy of other classes.

The main contributions of this work can be summarized as follows:

▪ The design and implementation of an efficient and effective approach for detecting botnet attacks in resource-constrained IoT devices by using the Fisher-score-based feature selection method and an optimized XGBoost classifier;
▪ Irrelevant features are discarded by minimizing the within-class distance and maximizing the between-class distance;
▪ A genetic algorithm is combined with an XGBoost classifier to learn a genetic-based extreme gradient boosting (GXGBoost) model and to optimize the classification model;
▪ The GXGBoost model is used to solve an imbalanced classification problem;
▪ The proposed approach is evaluated and tested using a public botnet dataset of IoT devices based on holdout and 10-fold cross-validation techniques;
▪ The proposed approach is compared with state-of-the-art works on the same botnet dataset.

The rest of the paper is prepared as follows. In Section 2, the research methods used in this study are introduced. Section 3 outlines the proposed approach. Section 4 describes the experiments and discussions, including a description of the dataset, evaluation metrics, experimental results, and comparisons with some recent related works. Finally, Section 5 presents the conclusions and directions for future work.

## 2. Background

In this section, we outline the different methods adopted to formulate and design the proposed approach.

### 2.1. Fisher Score

The Fisher score is a filtered feature selection method. It is an effective supervised method that has been widely used for various practical problems related to feature selection [46]. As a filter-type feature selection method, each feature is first evaluated, a feature score is given, and then the degree of that feature is determined based on the score [47]. When selecting a subset, these are sorted in descending order according to the score of each feature and according to the number of features contained in the subset. The corresponding number of features is selected to form the subset. The Fisher score [46] evaluation criteria can be formulated as follows:

(1) $$\;S_{F}\left(f_{i}\right)=\frac{\sum_{j=1}^{C}n_{j}{\left(\mu_{i,j}-\mu_{i}\right)}^{2}}{\sum_{j=1}^{C}n_{j}\sigma_{i,j}^{2}}\;$$

where $\mu_{i}$ represents the mean of the feature $\;f_{i}$; $n_{j}$ represents the number of samples in the *j*th class; $\;\mu_{i,j}$ and $\sigma_{i,j}\;$ are the mean and variance of the feature $f_{i}$ in the j class, respectively. Algorithm 1 lists the pseudocode of the fisher score method.
**Algorithm 1.** Pseudocode of Fisher score method.1. **Initialization**2. scores = [], index = {}, shapes = {};3. **Input**:4. Training_Set;5. **Begin**6. For c in num_classes:7.   index[c] = df_fisher[‘class’] == c;8.   shapes[c] = df_fisher[index[c]].shape [0];9. End for c10. For col in df_fisher.columns:11.   If col == ‘class’:12.   continue13.   num = 0; den = 0;14.   m = df_fisher[col].mean();15.   For c in num_classes:16.    num = num+ (shapes[c]/df_fisher.shape [0]) *  (m-df_fisher[index[c]][col].mean()).^2;17.    den = den + (shapes[c]/df_fisher.shape[0]) *  df_fisher[index[c]][col].var();18.  End for c19.  score = {‘feature’: col, ‘score’: num/den};20.  scores.append(score);21. End for col22. **Return** Training_Set with selected features;23. **End**

### 2.2. Genetic Algorithm (GA)

A genetic algorithm (GA) is a computational model that simulates the natural evolution of biological theory. It is a method that is used to search for an optimal solution by simulating the natural evolution process. The GA begins with a population that represents a potential set of solutions to a problem. The population consists of a certain number of individuals that are encoded by genes [48].

The GA generates an initial population of candidates. The easiest way to setup an initial population is to randomly generate a large number of “individuals” (individual genes) with upper and lower bounds. Then, it calculates the fitness of each individual in the current population. After this, it selects a certain number of individuals with the highest fitness as the parents of the next generation. Then, the selected parents are paired. The parents are used to recombine and produce offspring with a certain probability of random mutation, then the offspring join to form a new generation within the population [49]. The selected parents continue to produce offspring until the number of new groups reaches the upper limit, otherwise the new group becomes the current group. The main features of GA implemented in our approach are crossover and mutation features. These can perform two different roles. Practically, the crossover feature is a convergence operation that pulls the population towards a local maximum of accuracy. In contrast, the mutation feature is a divergence operation that occasionally breaks one or more individuals of a population out of the local maxima space and discovers a better maxima space. Since the GA aims to bring the population to convergence, crossover happens more frequently than a mutation, which only affects a few individuals in a population in a given generation.

### 2.3. Extreme Gradient Boosting (XGBoost)

The extreme gradient boosting (XGBoost) method is a kind of gradient boosting decision tree (GBDT) [50] technique, which can be used for both classification and regression problems. As described in [51], gradient boosting is an ensemble learning method that combines a set of weak classifiers $\;f_{i}\left(x\right)$ to form a strong classifier  F(x). Therefore, boosting methods have three elements [52]:

- A loss function that must be optimized, for example cross-entropy is used for classification problems and mean squared error is used for regression problems;
- A weak learner to make predictions, such as decision trees;
- An additive model, whereby multiple weak learners are added together to form a strong learner, which makes the target loss function extremely small.

The gradient boosting tries to correct the residuals of all the weak learners by adding new weak learners [50]. In the end, multiple learners are added together for the final prediction and the accuracy is higher than for a single learner. It is called gradient boosting because it uses a gradient descent algorithm to minimize the training loss when adding new models. In general, the implementation of gradient boosting is relatively slow, because each time a tree must be constructed and added to the entire model sequence. XGBoost is characterized by fast calculation speed and good model performance [53]. The objective function of XGBoost can be divided into the loss function + regular term as follows:

(2) Obj(Θ)=L(Θ)+Ω(Θ) 

The loss function can tell us how well the model fits the data and the regular term can penalize complex models and encourage simple models.

The XGBoost algorithm would improve the capabilities of predictive models. One of these improvements is through regularization, in which the XGBoost adds a regular term to the cost function to control the complexity of the model [54]. The regular term contains the number of leaf nodes in the tree and the sum of the squares of the L2 modulus of the score output on each leaf node. From a bias–variance tradeoff perspective, the regular term reduces the variation of the model, makes the learned model simpler, and prevents overfitting. This is also a feature of XGBoost that is superior to the traditional GBDT approach.

## 3. Proposed Approach

**Figure 2:**
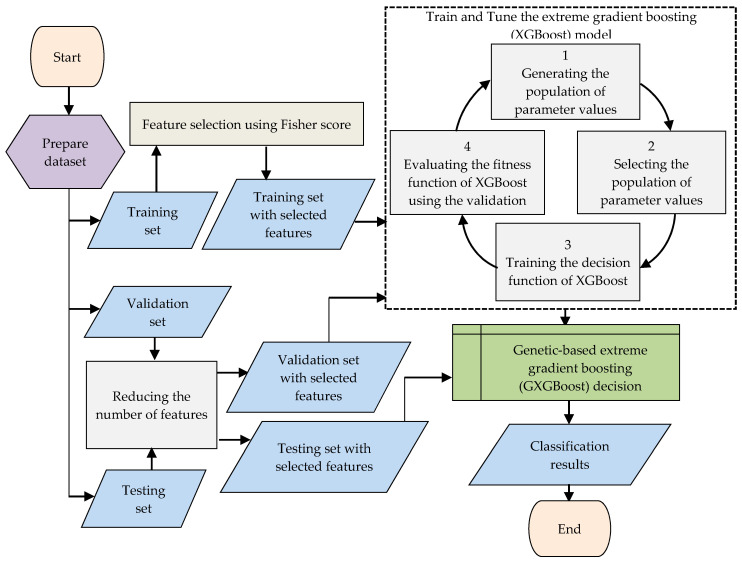
Flowchart of the proposed genetic-based extreme gradient boosting (GXGBoost) model and feature selection approach.

The proposed approach is based on a Fisher-score-based feature selection method and a genetic-based extreme gradient boosting (GXGBoost) model. GXGBoost is an effective ensemble model that was proposed in [51] to detect intrusion attacks in wireless sensors networks in which the features of the data traffic are quite small. The GXGBoost model uses the genetic algorithm to select the optimal values of model parameters to improve the accuracy of minority classes without affecting the overall accuracy of other classes. The proposed approach in this paper aims to improve the efficiency of the GXGBoost model to detect the intrusion attacks on data traffic and has high dimensionality of features in resource-constrained IoT devices. The Fisher score method is used for feature selection due to its ability to minimize the distance between features within classes and to maximize the distance between features between classes. In other words, the Fisher score is a representative filter-based feature selection method that plays an effective role in dimensionality reduction by minimizing within-class distance and maximizing between-class distance. Therefore, it is used to select the most important features and ignore irrelevant features in order to improve detection rates for botnet attacks in IoT devices. Figure 2 shows the main steps of the proposed approach. The approach starts with separating the dataset into training, validation, and testing sets.

The feature selection step is first applied on the training set using the Fisher score method. After this, the features of other validation and testing sets are filtered to contain only the selected features for training and testing of the GXGBoost model. Figure 2 illustrates the main method used to train the decision function of the XGBoost, which involves using the training set, and the method used to tune the values of its parameters, which involves evaluating its fitness function based on the validation set. The methods used to develop the GXGBoost model are described briefly in the previous subsections. More details and an explanation of the GXGBoost algorithm steps are given in [51]. Algorithm 2 describes the pseudocode of the GXGBoost model.
**Algorithm 2. Pseudocode of genetic-based extreme gradient boosting (GXGBoost) model.**1.**Initialization**2.minPopulation = 5;//Minimum population of GA3.maxPopulation = 10;//Maximum population of GA4.numIterations = 10;//Number of iterations to evaluate GA5.minNumEstimators =5, minLearningRate =0.1, lowMaxDepth =2;6.maxNumEstimators =50, maxLearningRate =0.4, highMaxDepth =20;7.**Input**8.TrainingSet, ValidationSet;9.**Begin**10.numPopulation = random.randint (minPopulation, maxPopulation);//Generating random number of population11.populationGXGBoost = [];12.For *i* in range (numPopulation):13.GXGBoostParameters = random.randint (minNumEstimators, minLearningRate, lowMaxDepth, maxNumEstimators, maxLearningRate, highMaxDepth);//Generating random values of GXGBoost’s parameters14.GXGBoostModel = generateGXGBoost (GXGBoostParameters);15.populationGXGBoost.append (GXGBoostModel);16.End for *i*17.maxAccuracy = 0;18.best_model = None;19.populationValidationAccuracy = [];20.For *i* in range (numIterations):21. For *j* in range (numPopulation):22. GXGBoostModel = populationGXGBoost [j];//Evaluating population23. validationAccuracy = evaluateGXGBoost (GXGBoostModel, TrainingSet, ValidationSet);24.populationValidationAccuracy.append (validationAccuracy);25.If validationAccuracy > maxAccuracy:26. maxAccuracy = validationAccuracy;27. bestModel = GXGBoostModel;28.End if29.End for
//Creating the new generations from the current best GXGBoostModel30.For *popIndex* in range (numPopulation):31. model_1 = populationGXGBoost [popIndex];32. model_1ValidationAccuracy = populationValidationAccuracy [popIndex];33. model_2 = bestModel;34. model_2ValidationAccuracy = maxAccuracy;
//Creating the new generations with crossovers35. newModel = crossoverGXGBoost (model_1, model_1ValidationAccuracy, model_2, model_2ValidationAccuracy);36. mutateGXGBoost (newModel);//Mutating the new generations37. populationGXGBoost [popIndex] = newModel;//Replace current model 38.End for39.End for 40.**Return** bestModel, maxAccuracy;41.**End**

## 4. Experiments and Discussion

The experiments in this research were conducted on a public dataset, named network-based IoT (N-BaIoT) dataset [31], to detect botnet attacks in data traffic for IoT devices. The N-BaIoT dataset will be described in Section 4.1. The experimental results will be presented and discussed in Section 4.2.

### 4.1. N-BaIoT Dataset

N-BaIoT is a public dataset used for detecting botnet attacks in IoT devices. It was created by Meidan et al. [31]. This dataset contains more than 5 million points of real traffic data used to address the unavailability of real public IoT botnets. It was collected from nine IoT devices attacked by two families of botnets from an isolated network. The two families of botnets are Bashlite and Mirai. They are the most common botnets in IoT devices and they have harmful capabilities. In this dataset, the number of instances is different for each attack in each device. The N-BaIoT dataset contains a set of files; each file has 115 features, in addition to the class labels “benign” or “transmission control protocol (TCP) attack” for binary classification. The TCP attacks are also divided into Mirai and Bashlite attacks for multiclass classification. The 115 features comprise aggregated statistics for each data point in the network raw streams, representing five time windows—1 min, 10 s, 1.5 s, 500 ms, and 100 ms, which are coded as L0.01, L0.1, L1, L3, and L5, respectively. These features are distributed within five major categories, as shown in Table 1.

**Figure 3:**
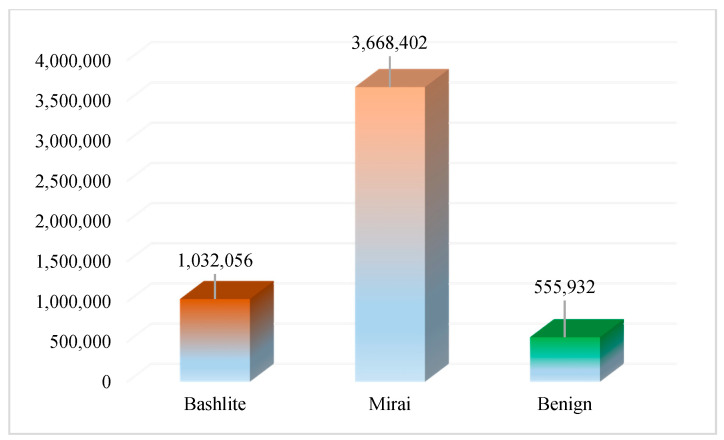
Number of instances in the dataset for Mirai, Bashlite, and benign classes.

For each category, the mean, packet size, packet count, and variance are calculated. For the socket and channel category, supplementary statistics are provided, such as the packet size correlation coefficient, magnitude, radius, and covariance. The distribution of class labels in the dataset is visualized in Figure 3.

**Figure 4:**
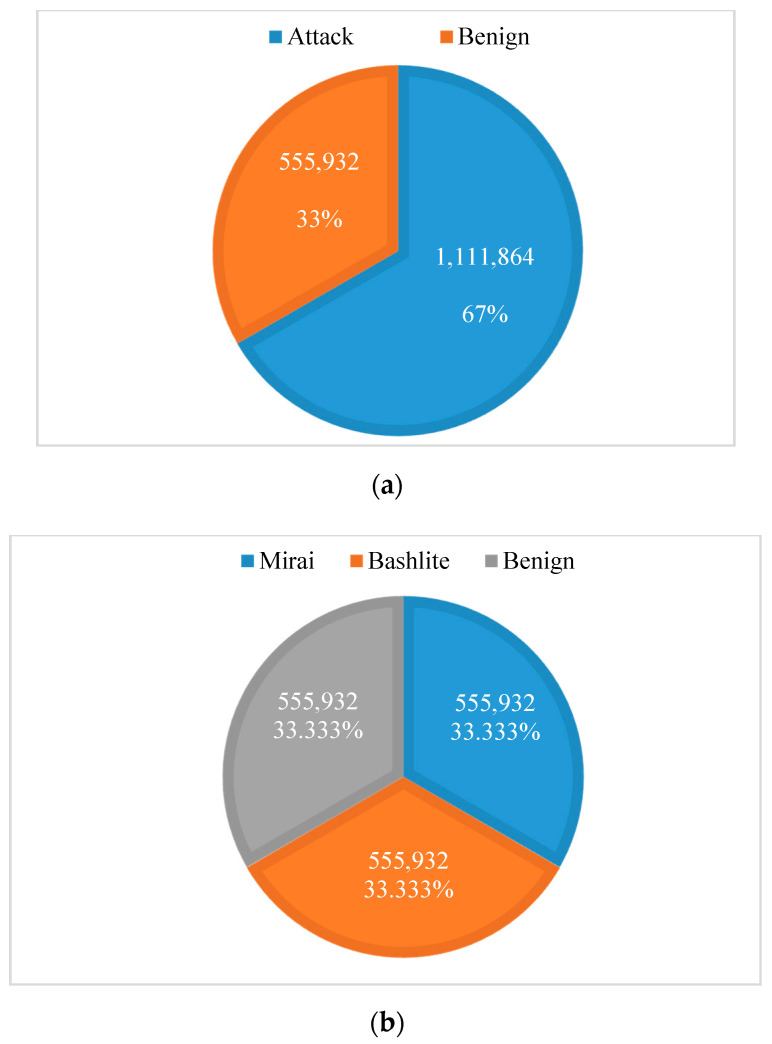
The distribution of instances in the dataset: (**a**) the distribution of instances according to the Mirai, Bashlite, and benign classes; (**b**) the distribution of instances according to attack and benign classes.

Due to the dataset being dominated by the Mirai class, and because we wanted to compare the “benign” class with other classes, the instances of Bashlite and Mirai classes were sampled at the “benign” class size to make them more balanced. Figure 4a shows the distribution of instances according to the Mirai, Bashlite, and benign classes in the dataset. Figure 4b illustrates the distribution of instances according to attack and benign classes in the dataset.

### 4.2. Experimental Results

The experimental results were figured out through the implementation of the proposed approach using Python programming language on a laptop with and Intel Core i7 2.2 GHz CPU, 32 GB RAM, and 64-bit Windows 10 operating system.

These results were assessed using a number of performance measures, including a confusion matrix; the numbers of true positive (*TP*) instances, true negative (*TN*) instances, false positive (*FP*) instances, and false negative (*FP*) instances; and the accuracy, precision, recall, and F1-scores. The confusion matrix is a table that represents the number of instances for each class that are correctly classified and the number of instances that are not correctly classified. One should note that the *TP, TN, FP,* and *FN* instances are obtained from the confusion matrix. The other performance measures can be defined using the following equations:

(3) $$Accuracy\;=\;\frac{\left(TP+TN\right)}{\left(TP+FP+TN+FN\right)}$$

(4) $$Precision\;=\;\frac{TP}{TP+FP}$$

(5) $$Recall=\;\frac{TP}{TP+FN}$$

(6) $$F1-\mathrm{score}\;=\;\frac{2\times \left(Recall\times Precision\right)}{\left(Recall+Precision\right)}$$

**Figure 5:**
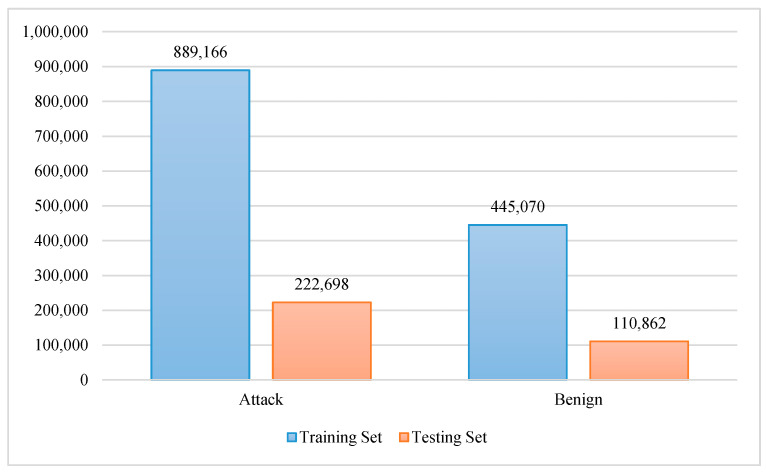
Number of instances in the training and testing sets for attack and benign classes.

**Figure 6:**
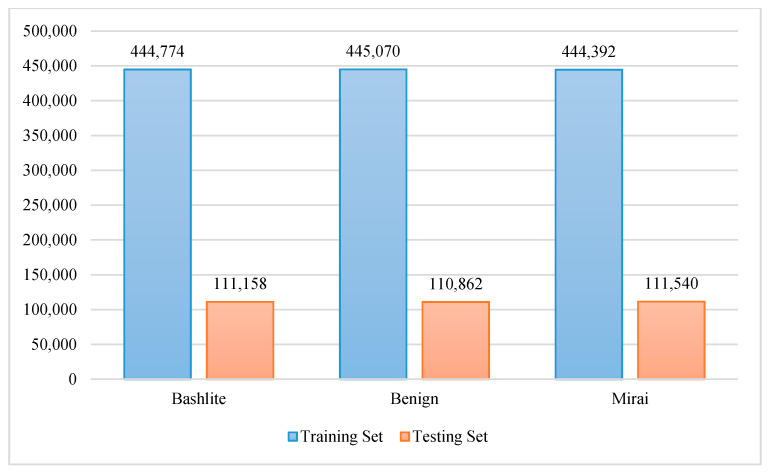
Number of instances in the training and testing sets for Mirai, Bashlite, and benign classes.

The testing sets for the experiments were obtained from the network-based IoT (BaIoT) dataset using holdout and 10-fold cross-validation techniques. In the holdout technique, the dataset is divided, with 80% being used for training and 20% being used for testing. Figure 5 and Figure 6 show the number of instances in the training and testing sets for binary and multiclass detection.

For the 10-fold cross-validation technique, the dataset is divided into 10 parts, one of which is used for testing, with the others being used for training. Regarding the training set for the holdout technique, 20% is used as a validation set to tune the parameters of the GXGBoost model.

All instances in the training, validation, and testing sets are normalized and then the Fisher score method is applied to the training set to rank the features in descending order based on their scores. Table 2 lists the features and their Fisher scores. In Table 1, the features are sorted in decreasing order based on their Fisher scores in order to target labels.

These scores are defined as the distances between the means of instances for each class label and each feature, which are divided by their variances. Hence, the Fisher score method ranks each feature independently according to Fisher criterion. From Table 1, we can see that the features “MI_dir_L0.01_weight”, “H_L0.01_weight”, “MI_dir_L0.01_mean”, “H_L0.01_mean”, and “MI_dir_L0.1_mean”, which have the numbers 13, 28, 14, 29, and 11, respectively, are the top five features. Consequently, we expect that these features will achieve the best results.

**Figure 7:**
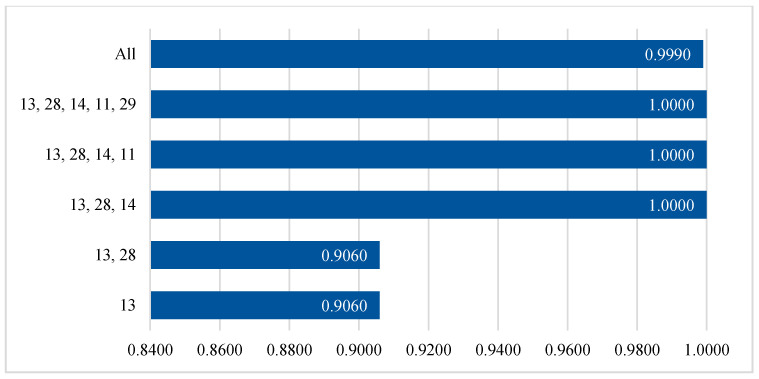
Accuracy results for the testing set for different numbers of features when detecting attack and benign classes.

The next step is the training process for the GXGBoost model, involving the training set and tuning or optimization using the GA on the validation set, as shown in Algorithm 2. The three most important parameters of the GXGBoost model are the learning rate, the max depth, and the number of estimators. These parameters are initialized with random values that represent the minimum and maximum values. The tuning process for the GXGBoost model tunes these parameters to have values of 0.2, 5, and 20, respectively. The other parameters are left to have default values. Figure 6 and Figure 7 show the F1-score results for different numbers of features when detecting attack and benign classes, and when detecting Mirai, Bashlite, and benign classes, respectively.

**Figure 8:**
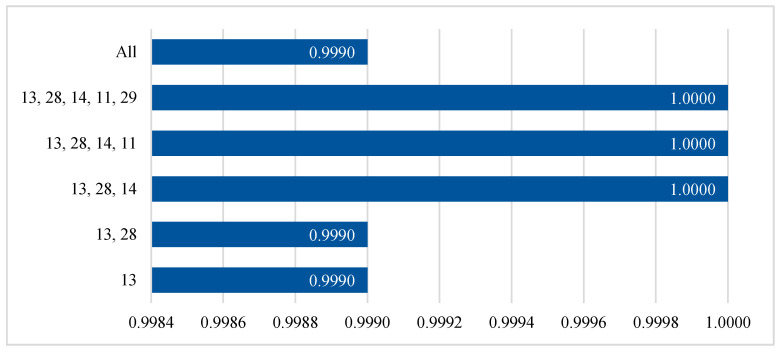
Accuracy results for the testing set with different numbers of features when detecting Mirai, Bashlite, and benign classes.

Figure 7 and Figure 8 show that feature numbers 13, 28, and 14, are the minimum numbers of features that achieved the highest weighted average F1-scores when detecting both the attack and benign classes, and detecting the Mirai, Bashlite, and benign classes.

**Figure 9:**
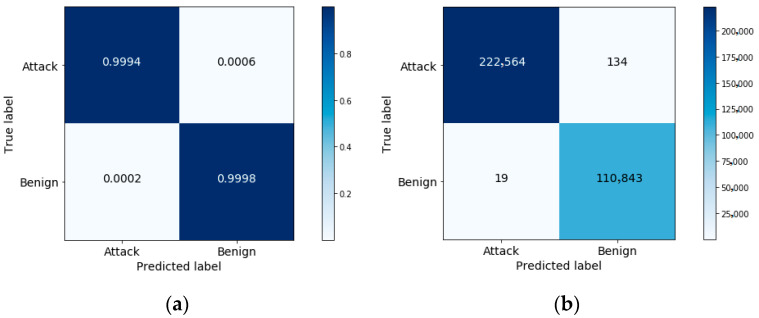
A confusion matrix for attack and benign classification: (**a**) with normalization; (**b**) without normalization.

**Figure 10:**
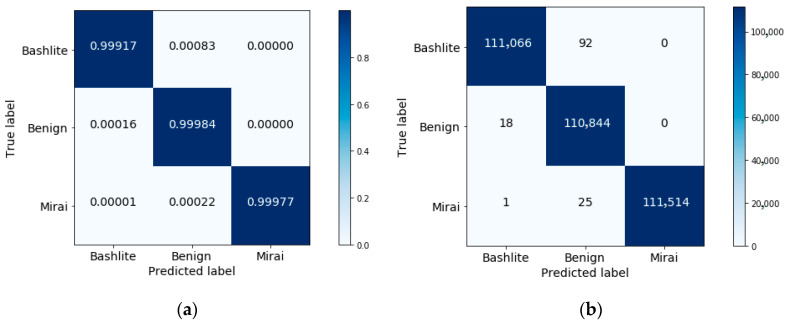
A confusion matrix for Mirai, Bashlite, and benign classification: (**a**) with normalization; (**b**) without normalization.

To explore this further, the confusion matrices using the selected three features are shown in Figure 9 and Figure 10. Table 3 and Table 4 list the results for the other performance measures.

In Figure 9b, we can see that the model correctly detects 222,564 instances out of 222,583 as “attack” class, which represents the TP measure, and incorrectly detects 19 instances out of 222,583 as “benign” class, which represents the FP measure. In addition, the model correctly detects 110,843 instances out of 110,977 as “benign” class, which represents the TN measure, and incorrectly detects 134 instances out of 110,977 as “attack” class, which represents the FN measure. To compute the TP rate, we divide the TP over the total true attack class number (222,564/(222,564 + 134)) to get 0.99940. To compute the FP rate, we divide the FP over the total true benign class number (19/(19 + 110,843)) to get 0.00017. From these results, we notice that the approach can achieve a low FP rate and a high TP rate when effectively differentiating attack patterns from benign data traffic.

From Figure 9 and Figure 10, as well as the results reported in Table 3 and Table 4, one can see that the performance attained using the selected features exceeds that achieved using all features.

To validate the results of the holdout technique for the proposed approach, we reported the results of the 10-fold cross-validation to detect the attack and benign classes using the selected features in Table 5, Table 6 and Table 7. Similarly, Table 8, Table 9 and Table 10 show the results of the 10-fold cross-validation for detecting Mirai, Bashlite, and benign classes based on the selected features.

Moreover, to evaluate the efficiency of the proposed approach, the execution time averages for training the GXGBoost model on 1,334,236 instances and testing it on 333,560 instances were computed using the three selected features and using all features, as shown in Table 11.

Table 11 demonstrates clearly that the average execution time taken to train and test the GXGBoost model using the selected features is significantly lower than the average execution time spent using all features. This confirms the efficiency and feasibility of the proposed approach for real-time systems in which the quantitative expression of time is needed to describe the detection response in such systems. This approach is suitable for systems with hard real-time constraints, whereby these systems must first detect the incoming attacks in a corresponding timeframe before they can do any damage. On the other hand, to compute the time complexity in order to construct the GXGBoost model, we use a big-O notation. Big-O notation is a mathematical notation, used in a computer science field to describe how the run time of an algorithm scales according to the size of input variables. Thus, we assumed that we had *n* populations and *n* iterations in the worst case. In addition, the time complexity for building the gradient boosting model is  O(dn), where d represents the number of features and n is the number of data samples [51]. Therefore, the time complexity for constructing the GXGBoost model is $O\left(dn^{3}\right)$, a cubic polynomial time. Because the proposed approach reduced the number of features, d, to three, this made the running time more efficient when detecting botnet attacks on IoT devices, which have limited computing resources.

Table 12 and Table 13 summarize and introduce a comparison between the proposed approach and the recent related works on the N-BaIoT dataset. Table 12 compares the results with related works on the detection of attack and benign classes. Table 13 compares the results of the proposed work with related works on detecting Mirai, Bashlite, and benign classes.

From Table 12 and Table 13, one can obviously see that the proposed approach outperforms the related works in detecting botnet attacks on IoT devices using three features. Although the deep autoencoder-based approach in [55] achieved a competitive accuracy result for detecting attack and benign classes using three features, our proposed approach attained the highest accuracy result, with the advantage that it can be executed once to select the best features, compared to the autoencoder-based feature reduction approach, which is executed in each run to reduce the features, adding an extra overload to the detection task. Another disadvantage of the autoencoder-based approach is the large number of parameters that need to be optimized and initialized in the training process. Accordingly, the proposed approach exhibits many advantages that improve the effectiveness and efficiency of botnet attack detection for resource-constrained IoT devices.

### 4.3. Statistical Tests

In this subsection, we report the results of the statistical test conducted to validate the obtained accuracy results. This test is meant to judge the significance of the obtained results and to ensure the fairness of the comparison with the relevant methods. To conduct this statistical test, a one-sample t-test was used to compare the accuracy results of the 10-fold cross-validation with the accuracy results of comparative methods. The one-sample t-test is a statistical parametric analysis measure that compares the mean of random samples to a hypothesized mean value and tests for a deviation from that hypothesized value. Using the one-sample t-test, we formulated the hypotheses for the statistical analysis as follows:

• *Null hypothesis*: The mean of the accuracy results for 10-fold cross-validation of the proposed approach is equal to the hypothesized accuracy value, which is 99.96%.
• *Alternative hypothesis*: The mean of accuracy results for the 10-fold cross-validation of the proposed approach is not equal to the hypothesized accuracy value, which is 99.96%.

After conducting the analysis using SPSS software, the test results were obtained, which are documented in Table 14 and Table 15. Table 14 shows the one-sample t-test results for the accuracy of attack and benign classification using a 10-fold cross-validation technique. In addition, Table 15 illustrates the one-sample t-test results for the accuracy of classification of the Mirai, Bashlite, and benign classes.

In Table 14 and Table 15, the degrees of freedom (DF) represent the amount of information in the data sample of size *n*-1 that can be provided to compute the variability of the estimates for the parameter values of an unknown population. Thus, the one-sample t-test applies a t-distribution with *n*-1 DF. As we used a 10-fold cross-validation technique in our statistical analysis test, the sample size was 10; one of the samples was used to estimate the mean of the accuracy results and the remaining nine DF were used to estimate the variability.

From the results of the one-sample *t*-test shown in Table 14, we can see that the *p*-value is 1 for the accuracy of the attack and benign classification. Moreover, as can be seen in Table 15, the *p*-value reaches 0.343 for the accuracy of the Mirai, Bashlite, and benign classification. For both cases, the *p*-value is greater than 0.05. Therefore, we can accept the null hypothesis for the proposed approach for classifying benign and IoT botnet attack classes. Consequently, the mean of the accuracy results for the proposed approach for all test sets of the 10-fold technique is equal to the hypothesized accuracy value, which is 99.96%. This result was used to compare the approach with recent related works. This means that the distribution of accuracies for all test sets is almost the same and there is no statistically significant differences between them, which confirms the stability and effectiveness of the proposed approach against the overfitting problem.

## 5. Conclusions and Future Work

IoT devices and technology have been widely used in many network-based applications in different fields. Increasing the number of vulnerable or unprotected IoT devices makes the system easily compromised by attackers through a set of botnets, enabling a large-scale cyber-attack. To solve this issue effectively and efficiently, we proposed an IoT botnet attack detection approach using a Fisher-score-based feature selection method with a genetic-based extreme gradient boosting (GXGBoost) model. The Fisher score is a representative filter-based feature selection method that is used to select significant features and reduce irrelevant features by minimizing the within-class distance and maximizing the between-class distance. GXGBoost is an optimized model that is applied for effective classification of IoT botnet attacks. Several experiments were performed on a public botnet dataset of IoT devices using a holdout testing technique. The evaluation results showed that the proposed approach has a high detection rate using only three out of 115 data traffic features, improving the efficiency of IoT botnet attack detection. Even though the ability of the proposed approach to find the best values for XGBoost’s parameters in a short computation time is known, one of its limitations is that there is no way to confirm that these parameter values reach the global optima. Another limitation is related to the sensitivity of the GA used for the initial population and its randomness, which may lead to the inability to explore the search space of the solutions. In future work, we will use different optimization algorithms in the proposed approach to tune XGBoost’s parameters for detection of IoT botnet attacks.

## Figures and Tables

**Table 1:** Description of the dataset features.

Category	Description
host-internet protocol (host-IP)	This category is coded as H. It represents the statistical value of the features for the traffic coming from a specific IP address.
host-medium access control and internet protocol (host-MAC&IP)	This category is coded as MI. It represents the statistical value of the features for the traffic coming from the same IP and MAC.
channel	This category is coded as HH. It represents the statistical value of the features for the traffic between particular hosts.
socket	This category is coded as HpHp. It represents the statistical value of the features for the traffic between particular hosts that contain ports.
network-jitter	This category is coded as HH_jit. In channel communication, the statistical value of the features related to the time intervals between packets is included in this category.

**Table 2:** The features with their Fisher scores.

F. No.	Feature Name	Fisher Score	F. No.	Feature Name	Fisher Score	F. No.	Feature Name	Fisher Score
13	MI_dir_L0.01_weight	1.03	96	HpHp_L1_mean	0.30	38	HH_L3_weight	0.04
28	H_L0.01_weight	1.03	61	HH_L0.01_std	0.28	69	HH_jit_L3_weight	0.04
14	MI_dir_L0.01_mean	0.77	112	HpHp_L0.01_magnitude	0.27	31	HH_L5_weight	0.04
29	H_L0.01_mean	0.77	105	HpHp_L0.1_magnitude	0.27	66	HH_jit_L5_weight	0.04
11	MI_dir_L0.1_mean	0.75	84	HpHp_L5_magnitude	0.27	102	HpHp_L0.1_weight	0.04
26	H_L0.1_mean	0.75	91	HpHp_L3_magnitude	0.27	95	HpHp_L1_weight	0.04
15	MI_dir_L0.01_variance	0.73	98	HpHp_L1_magnitude	0.27	88	HpHp_L3_weight	0.04
30	H_L0.01_variance	0.73	62	HH_L0.01_magnitude	0.26	109	HpHp_L0.01_weight	0.04
8	MI_dir_L1_mean	0.72	55	HH_L0.1_magnitude	0.26	81	HpHp_L5_weight	0.04
23	H_L1_mean	0.72	48	HH_L1_magnitude	0.25	113	HpHp_L0.01_radius	0.03
12	MI_dir_L0.1_variance	0.71	34	HH_L5_magnitude	0.25	99	HpHp_L1_radius	0.03
27	H_L0.1_variance	0.71	41	HH_L3_magnitude	0.25	80	HH_jit_L0.01_variance	0.03
9	MI_dir_L1_variance	0.70	54	HH_L0.1_std	0.17	106	HpHp_L0.1_radius	0.03
24	H_L1_variance	0.70	79	HH_jit_L0.01_mean	0.16	77	HH_jit_L0.1_variance	0.03
5	MI_dir_L3_mean	0.64	76	HH_jit_L0.1_mean	0.16	92	HpHp_L3_radius	0.03
20	H_L3_mean	0.64	73	HH_jit_L1_mean	0.14	100	HpHp_L1_covariance	0.03
6	MI_dir_L3_variance	0.61	70	HH_jit_L3_mean	0.14	85	HpHp_L5_radius	0.03
21	H_L3_variance	0.61	67	HH_jit_L5_mean	0.14	93	HpHp_L3_covariance	0.03
10	MI_dir_L0.1_weight	0.61	111	HpHp_L0.01_std	0.14	86	HpHp_L5_covariance	0.03
25	H_L0.1_weight	0.61	59	HH_L0.01_weight	0.13	50	HH_L1_covariance	0.02
2	MI_dir_L5_mean	0.58	78	HH_jit_L0.01_weight	0.13	58	HH_L0.1_pcc	0.02
17	H_L5_mean	0.58	104	HpHp_L0.1_std	0.12	94	HpHp_L3_pcc	0.02
3	MI_dir_L5_variance	0.51	47	HH_L1_std	0.10	87	HpHp_L5_pcc	0.02
18	H_L5_variance	0.51	40	HH_L3_std	0.09	43	HH_L3_covariance	0.02
1	MI_dir_L5_weight	0.48	33	HH_L5_std	0.09	108	HpHp_L0.1_pcc	0.02
16	H_L5_weight	0.48	65	HH_L0.01_pcc	0.08	36	HH_L5_covariance	0.02
4	MI_dir_L3_weight	0.45	97	HpHp_L1_std	0.08	57	HH_L0.1_covariance	0.02
19	H_L3_weight	0.45	90	HpHp_L3_std	0.07	101	HpHp_L1_pcc	0.02
7	MI_dir_L1_weight	0.45	83	HpHp_L5_std	0.07	107	HpHp_L0.1_covariance	0.02
22	H_L1_weight	0.45	63	HH_L0.01_radius	0.06	74	HH_jit_L1_variance	0.01
60	HH_L0.01_mean	0.30	56	HH_L0.1_radius	0.05	114	HpHp_L0.01_covariance	0.01
53	HH_L0.1_mean	0.30	49	HH_L1_radius	0.05	51	HH_L1_pcc	0.00
110	HpHp_L0.01_mean	0.30	52	HH_L0.1_weight	0.05	44	HH_L3_pcc	0.00
46	HH_L1_mean	0.30	75	HH_jit_L0.1_weight	0.05	37	HH_L5_pcc	0.00
39	HH_L3_mean	0.30	42	HH_L3_radius	0.05	64	HH_L0.01_covariance	0.00
82	HpHp_L5_mean	0.30	35	HH_L5_radius	0.05	71	HH_jit_L3_variance	0.00
32	HH_L5_mean	0.30	45	HH_L1_weight	0.04	68	HH_jit_L5_variance	0.00
89	HpHp_L3_mean	0.30	72	HH_jit_L1_weight	0.04	38	HH_L3_weight	0.04
103	HpHp_L0.1_mean	0.30	115	HpHp_L0.01_pcc	0.04	69	HH_jit_L3_weight	0.04

**Note:** The F. No. in the column title of the table represents the feature number.

**Table 3:** Results for performance evaluation measures when detecting attack and benign classes using the selected features numbers 13, 28, and 14.

Class Name	Precision	Recall	F1-Score
**Attack**	0.999	1.000	1.000
**Benign**	1.000	0.999	0.999
**Weighted average**	1.000	1.000	1.000
**Accuracy**	1.000		

**Table 4:** Results for performance evaluation measures for Mirai, Bashlite, and benign classification using the selected features numbers 13, 28, and 14.

Class Name	Precision	Recall	F1-Measure
**Bashlite**	0.999	1.000	1.000
**Benign**	1.000	0.999	0.999
**Mirai**	1.000	1.000	1.000
**Weighted average**	1.000	1.000	1.000
**Accuracy**	1.000		

**Table 5:** Precision results of the 10-fold cross-validation for attack and benign classifications using the selected feature numbers.

Fold No.	Precision		
	Attack	Benign	Weighted Avg.
1	0.9998	0.9988	0.9995
2	0.9999	0.999	0.9996
3	0.9999	0.9991	0.9997
4	0.9998	0.9992	0.9996
5	0.9999	0.9989	0.9996
6	0.9999	0.9987	0.9995
7	0.9999	0.9991	0.9996
8	0.9999	0.9989	0.9996
9	0.9999	0.999	0.9996
10	0.9999	0.9988	0.9995
Average (Avg.)	0.99988	0.99895	0.99958
Standard deviation (Std.)	0.00004	0.00016	0.00006

**Table 6:** Recall results of the 10-fold cross-validation for attack and benign classifications using the selected feature numbers.

Fold No.	Recall		
	Attack	Benign	Weighted Avg.
1	0.9994	0.9997	0.9995
2	0.9995	0.9997	0.9996
3	0.9995	0.9999	0.9997
4	0.9996	0.9997	0.9996
5	0.9995	0.9997	0.9996
6	0.9994	0.9999	0.9995
7	0.9996	0.9998	0.9996
8	0.9995	0.9997	0.9996
9	0.9995	0.9997	0.9996
10	0.9994	0.9998	0.9995
Average (Avg.)	0.99949	0.99976	0.99958
Standard deviation (Std.)	0.00007	0.00008	0.00006

**Table 7:** F1-scores and accuracy results of the 10-fold cross-validation for attack and benign classifications using the selected feature numbers.

Fold No.	F1-Score			Accuracy
	Attack	Benign	Weighted Avg.	
1	0.9996	0.9993	0.9995	0.9995
2	0.9997	0.9994	0.9996	0.9996
3	0.9997	0.9995	0.9997	0.9997
4	0.9997	0.9994	0.9996	0.9996
5	0.9997	0.9993	0.9996	0.9996
6	0.9996	0.9993	0.9995	0.9995
7	0.9997	0.9995	0.9996	0.9996
8	0.9997	0.9993	0.9996	0.9996
9	0.9997	0.9994	0.9996	0.9996
10	0.9997	0.9993	0.9995	0.9995
Average (Avg.)	0.99968	0.99937	0.99958	0.99958
Standard deviation (Std.)	0.00004	0.00008	0.00006	0.00006

**Table 8:** Precision results of the 10-fold cross-validation for Mirai, Bashlite, and benign classifications using the selected feature numbers.

Fold No.	Precision			
	Bashlite	Benign	Mirai	Weighted Avg.
1	0.9997	0.9989	1.0000	0.9995
2	0.9998	0.9990	0.9999	0.9996
3	0.9998	0.9992	1.0000	0.9997
4	0.9997	0.9992	1.0000	0.9996
5	0.9998	0.9989	1.0000	0.9996
6	0.9999	0.9988	0.9999	0.9996
7	0.9998	0.9991	1.0000	0.9996
8	0.9998	0.9990	1.0000	0.9996
9	0.9997	0.9991	1.0000	0.9996
10	0.9998	0.9989	1.0000	0.9996
Average (Avg.)	0.99978	0.99901	0.99998	0.9996
Standard deviation (Std.)	0.00006	0.00014	0.00004	0.00005

**Table 9:** Recall results of the 10-fold cross-validation for Mirai, Bashlite, and benign classifications using the selected feature numbers.

Fold No.	Recall			
	Bashlite	Benign	Mirai	Weighted Avg.
1	0.9991	0.9997	0.9999	0.9995
2	0.9991	0.9997	0.9999	0.9996
3	0.9994	0.9998	0.9998	0.9997
4	0.9993	0.9997	0.9999	0.9996
5	0.9991	0.9998	0.9998	0.9996
6	0.9990	0.9999	0.9998	0.9996
7	0.9993	0.9998	0.9999	0.9996
8	0.9991	0.9998	0.9998	0.9996
9	0.9992	0.9997	0.9999	0.9996
10	0.9990	0.9998	0.9998	0.9996
Average (Avg.)	0.99916	0.99977	0.99985	0.9996
Standard deviation (Std.)	0.00013	0.00007	0.00005	0.00005

**Table 10:** F1-scores and accuracy results of the 10-fold cross-validation for Mirai, Bashlite, and benign classifications using the selected feature numbers.

Fold No.	F1-Score				Accuracy
	Bashlite	Benign	Mirai	Weighted avg.	
1	0.9994	0.9993	0.9999	0.9995	0.9995
2	0.9995	0.9994	0.9999	0.9996	0.9996
3	0.9996	0.9995	0.9999	0.9997	0.9997
4	0.9995	0.9994	0.9999	0.9996	0.9996
5	0.9994	0.9994	0.9999	0.9996	0.9996
6	0.9994	0.9993	0.9999	0.9996	0.9996
7	0.9995	0.9995	0.9999	0.9996	0.9996
8	0.9995	0.9994	0.9999	0.9996	0.9996
9	0.9995	0.9994	1.0000	0.9996	0.9996
10	0.9994	0.9994	0.9999	0.9996	0.9996
Average (Avg.)	0.99947	0.9994	0.99991	0.9996	0.9996
Standard deviation (Std.)	0.00007	0.00007	0.00003	0.00005	0.00005

**Table 11:** Average times taken for the GXGBoost model using 1,334,236 instances of the training set to detect Mirai, Bashlite, and benign classes from 333,560 instances of the testing set.

Features Size	Avg. Training Time (Sec.)	Avg. Testing Time (Sec.)
**Three selected features**	37.496	0.254
**All features**	545.040	4.208

**Table 12:** Comparison of results with related works for attack and benign classification tasks.

Ref.	Year	Approach	No. of Features	Accuracy
[55]	2019	Deep Autoencoder	3	99.91%
[56]	2018	Entropy support vector machine (SVM)	3	83.37%
[56]	2018	Variance Isolation Forest	3	62.29%
This work	2020	Fisher Score + GXGBoost Model	3	**99.96%**

**Table 13:** Comparison of results with related works for Mirai, Bashlite, and benign classification tasks.

Ref.	Year	Approach	No. of Features	Accuracy
[55]	2019	Deep Neural Network	3	86.63%
[57]	2018	Decision Tree	3	98.51%
[57]	2018	k-nearest neighbors (k-NN)	3	97.24%
This work	2020	Fisher Score + GXGBoost Model	3	**99.96%**

**Table 14:** One-sample t-test results for the accuracy of attack and benign classification using a 10-fold cross-validation technique.

	Test Value = 99.96%					
	t	DF	Sig. (2−Tailed)	Mean Difference	95% Confidence Interval of the Difference	
					Lower	Upper
Accuracy (%)	0.000	9	1.000	0.00000	−0.0034	0.0034

**Table 15:** One-sample t-test results for the accuracy of Mirai, Bashlite, and benign classification using a 10-fold cross-validation technique.

	Test Value = 99.96%					
	t	DF	Sig. (2−Tailed)	Mean Difference	95% Confidence Interval of the Difference	
					Lower	Upper
Accuracy (%)	−1.000	9	0.343	−0.00200	−0.0065	0.0025
